# Repetitive Transcranial Magnetic Stimulation in Severe OCD: A Case Report

**DOI:** 10.1192/j.eurpsy.2025.1708

**Published:** 2025-08-26

**Authors:** C. Chen Yee

**Affiliations:** General psychiatry, Institute of Mental Health, Singapore , Singapore

## Abstract

**Introduction:**

Obsessive-compulsive disorder (OCD) is characterized by recurrent intrusive distressing thoughts, images, or urges (obsessions), and/or by behavioral acts (compulsions) that the individual feels driven to perform. In patients with severe functional impairment, a combination of selective serotonin reuptake inhibitors (SSRIs) or chlomipramine and cognitive behavioural therapy is recommended. Evidence for use of repetitive transcranial magnetic stimulation (rTMS) as an adjunct in severe, medication-resistant cases of OCD is accumulating.

**Objectives:**

We present a case of severe OCD in a patient who responded poorly to SSRIs, and who was trialed on rTMS, with marked improvement.

**Methods:**

Details of the case were described. Information was gathered based on medical records.

**Results:**

Patient T was a 34-year-old Chinese male, with a drug allergy to clomipramine, who was diagnosed with severe OCD in 2017, when he presented to the Institute of Mental Health for excessive anxiety with checking behaviour. He was maintained on fluoxetine 60mg OM and aripiprazole 10mg OM, from 2017 to 2020. He completed a course of rehabilitation during admission, as well as empowerment and job training programs in the outpatient setting. He still had residual symptoms of excessive checking and would take a long time to complete activities of daily living such as eating or showering. He then defaulted medications and follow up from 2020 onwards. In 2024, he was brought in by his father to the emergency services for an infected foot wound due to poor self-care, as well as loss of weight from only eating one meal a day, due to his excessive checking behaviour. He would take 5 hours to finish a meal and 4 hours a day showering. He had also stopped speaking to anyone for 3 years. In the ward, he was switched to paroxetine 40mg OM as he had urinary incontinence on fluoxetine and weight gain on aripiprazole. However, improvement with paroxetine was minimal and the patient was still taking 2 hours each for showering and meals. The decision was made to give him a trial of rTMS. Drug treatment remained unchanged during the rTMS trial. Prior to rTMS, his Yale-Brown Obsessive-Compulsive Scale (YBCOS) was 30 (severe OCD).

After 30 sessions of rTMS, his obsessions and compulsions were significantly reduced, with a YBCOS of 17. He was able to finish his meals and shower for less than 30 minutes each. He was also more participative in ward activities with other patients, such as playing ping pong. He also showed interest in self-care by asking questions about his infected foot wound and prevention strategies.

**Conclusions:**

In this case, there was a need for early treatment to address the risk of self-neglect posed by the patient’s obsessive checking behaviour. rTMS is a safe and efficient treatment for patients suffering from refractory OCD. Further research is needed to optimize rTMS protocols and evaluate the long-term efficacy of rTMS for OCD.

**Disclosure of Interest:**

None Declared

